# The American Pharmaceutical Association

**Published:** 1877-10-20

**Authors:** 


The American Pharmaceutical Association.–The American Parmaceutical Assotion which held its late meeting in Toronto, appointed Atlanta, Georgia, as the next place of meeting, which will be on the first Tuesday of September, 1878, 3 o'clock p. m. Mr. J. W. Rankin, of Atlanta, was elected local Secretary for purposes of correspondence, &c.

